# Characteristics and experiences of peer counsellors in urban Dhaka: a structured interview study

**DOI:** 10.1186/s13006-019-0240-y

**Published:** 2019-11-06

**Authors:** Seema Mihrshahi, Hannah Tait, Rukhsana Haider, Gulshan Ara, Iqbal Kabir, Michael J. Dibley

**Affiliations:** 1 Sydney School of Public Health, Faculty of Medicine and Health, University of Sydney, Sydney, NSW Australia; 2 School of Public Health, University of Queensland, Herston, Queensland Australia; 3 Training and Assistance for Health and Nutrition Foundation (TAHN), Dhaka, Bangladesh; 4 International Centre for Diarrhoeal Diseases Research, Bangladesh (ICDDR,B), Dhaka, Bangladesh

## Abstract

**Background:**

Interventions to promote breastfeeding are the cornerstone of efforts to reduce childhood illness and death from undernutrition. Evidence suggests that one of the most effective strategies to increase breastfeeding is through peer counsellors. However, the experiences of peer counsellors has not been studied in depth. This study aimed to collect and report the experiences of peer counsellors participating in an intervention study to improve breastfeeding in urban Dhaka, Bangladesh.

**Methods:**

Peer counsellors underwent a 10 day training course in May 2013 which included practical sessions on position and attachment and common difficulties with breastfeeding. Home visits were conducted with new mothers and performance of peer counsellors was monitored by senior breastfeeding counsellors. The number of supervised home visits needed to achieve a satisfactory level of competency was recorded. Demographic data were collected and a structured interview was performed in the first six months of the project (May–September 2013). One structured interview per peer counsellor was conducted by the project manager at the project site office to gain understanding of their experiences in counselling mothers. The interview included some open-ended questions on specific aspects of the training that they found useful, challenges faced, and whether they developed close friendships with the mothers that they were counselling.

**Results:**

Seventeen peer counsellors with an average age of 31 years (SD 6.8) and at least six years of schooling participated in the study. All peer counsellors were satisfied with their role and with the training that they received, and most felt that they were able to deal with common breastfeeding problems. The peer counsellors reported that building a personal rapport and establishing a peer-to-peer relationship was most important in supporting mothers to breastfeed. Common challenges included interruption of sessions by relatives/children, as well as mothers being too busy for the visits.

**Conclusion:**

In future peer counselling for breastfeeding projects, more focus could be placed on the communications aspects of the training, especially in how to deal with non-supportive family members and managing interruptions effectively, as well as how to motivate and engage busy new mothers.

## Background

It has been estimated that nearly half of all deaths among children under 5 are linked to undernutrition [1]. Undernutrition is highly prevalent in developing countries with approximately 52 million children under 5 wasted, and 155 million children stunted [2]. Evidence suggests that major improvements in nutrition can be made through large-scale nutrition-sensitive programmes that address key underlying determinants of nutrition and by enhancing the coverage and effectiveness of nutrition-specific interventions and programs. Interventions include breastfeeding promotion and education on appropriate complementary feeding [3, 4]. In low-and middle-income countries about half of all diarrhoea episodes and a third of respiratory infections could be avoided by breastfeeding. In ambitious scale up models, breastfeeding promotion was estimated to be able to prevent the third largest number of child deaths (after pneumococcal vaccine and case management of neonatal infections), approximately 250,000 by 2025 [5]. Further to the immune protection that reduces the risk of infection, there are extensively documented social, developmental, economic and environmental benefits of breastfeeding for both the infant and mother [6, 7].

Despite this evidence, interventions to promote breastfeeding have poor coverage throughout many low and middle income countries [5] and globally only 37% of children younger than 6 months of age are exclusively breastfed. In Bangladesh, exclusive breastfeeding rates for infants 0–5 months have increased from 43% in 2007 to 64.1% in 2011 and back to 55% in 2014 [8]. Hospital initiatives, such as the Baby Friendly Hospital Initiative, can increase rates of early breastfeeding initiation [9]. However, in Bangladesh, where about 60–70% of births are home deliveries [8, 10], hospital based programmes have limited success. Evidence suggests that the most effective way to achieve increased initiation rates as well as exclusive breastfeeding is through the use of peer counselling networks [11, 12].

A systematic review of randomised or quasi-randomised controlled trials assessed the impact of breastfeeding promotion strategies [13]. In the studies conducted in low and middle income countries, the effect of breastfeeding promotion was a significant six-fold increase in exclusive breastfeeding prevalence [13]. An important finding of the review was that for improving rates of exclusive breastfeeding at 6 months, lay support had a significant impact, while education alone was ineffective. This has also been confirmed in a recent systematic review and meta-analysis of 47 studies [14]. Community based peer support for mothers in low and middle income countries was effective in increasing the rate of initiation of breastfeeding within the first hour of life, decreased the practice of prelacteal feeding and increased the duration of exclusive breastfeeding, particularly for infants aged 3–6 months [14].

Trials using face-to-face home visits conducted in Mexico [15], Bangladesh [12], Sub-Saharan Africa [11] and India [16] were successful in improving exclusive breastfeeding; compared to an intervention conducted via telephone in Malaysia [17] which was not as successful in improving exclusive breastfeeding at 4 and 6 months.

Developing enabling environments is crucial in assisting mothers to reach their breastfeeding goals by overcoming common barriers and complications [12, 18]. A review article showed the importance of the peer counsellor’s role in communicating breastfeeding knowledge in a socially and culturally specific way [19]. A recent study [20], is the only known study published to deal exclusively with the challenges faced by breastfeeding peer counsellors in low income contexts.

The aim of this study was to explore and describe the experiences of peer counsellors in supporting mothers to exclusively breastfeed in urban Dhaka. The study will help to document the barriers and successes of the peer counsellors and contribute towards future interventions to improve infant feeding.

## Methods

### Study design and intervention

The study was a community-based cluster randomised controlled trial using peer counsellors to improve infant and young child feeding outcomes, based in urban Dhaka. Primary outcomes of the study were to reduce the prevalence of stunting in children at 18 months; secondary outcomes were to improve breastfeeding and complementary feeding practices. Mothers received a total of 13 visits from peer counsellors, commencing in the antenatal period and continuing until 18 months of age. The mothers in the control group received standard care without the involvement of any peer support.

### Peer counsellor selection and training

Peer counsellors were selected based on a motivation to work, personal experience with breastfeeding, a minimum level of 6 years of schooling, and residence in the Mirpur area of Dhaka. In May 2013 the peer counsellors were trained over 10 days using the WHO/UNICEF breastfeeding counselling course previously simplified and adapted for use by the *Training and Assistance for Health and Nutrition Foundation (TAHN)* in a local context [12]. The training was completed at the project site office which was based in Mirpur, Dhaka close to the area where the peer counsellors lived. Counselling skills were taught by demonstration and role play, including listening to mothers, learning about their difficulties, assessing the position and attachment of the baby during breastfeeding, building mothers’ confidence, giving support, and providing relevant information and practical help when required. In terms of expectations of the project, peer counsellors were expected to work for a maximum of 4 h per day and visit at least one mother per day and record the times that they worked in order to calculate payments. This included times needed for travelling, phone calls and scheduling visits. Peer counsellors received refresher training every two months and were paid approximately BDT 4200 per month (AU$72 or USD $50 as of August 2019), which included a budget for mobile phone use.

### Data collection and analysis

Data collection occurred from May–September 2013 and data from 17 peer counsellors’ experiences were collected as follows. The first phase was to collect monitoring and performance data on the trained peer counsellors by the senior breastfeeding counsellors, the second phase was to collect demographic information and the third phase was to administer the structured interviews with some open-ended questions to the peer counsellors, to gain an understanding of their experience in working with mothers.

#### Phase 1

After the training course, peer counsellors performed approximately 10–20 visits to lactating mothers under the supervision of senior counsellors. Their performance was monitored at every session using an evaluation form (shown in Additional file 1). Peer counsellors were rated on a total of 15 practices/skills using a 5 point Likert scale from “none/inadequate” to “excellent”. The standard of practice was met when they achieved a rating of at least 4 “strong competency skills” for each of the 15 practices. After competency was established, peer counsellors were then allowed to complete sessions privately with mothers. The number of sessions needed by each peer counsellor to achieve competency were recorded.

#### Phase 2

Demographic information (including age, marital status, education level and work history) and details of feeding practices of the peer counsellors were collected through a questionnaire by trained research assistants at the project site office. Questions were adapted from those used in the Bangladesh Demographic and Health Survey [8] and this ensured the questions had been previously validated.

#### Phase 3

A structured interview was given and peer counsellors were asked to recount the challenges and experiences they were facing in their position. The questions used in the structured interview are given in Additional file 2. The answers were transcribed and then translated into English. Structured interviews included specific questions such as “how many visits do you perform each day” and also open-ended questions such as “what steps do you take when you are unsure of how to deal with a problem” and “what effects your ability to perform your job”. The interviews were administered by the project manager at the project site office.

### Coding and data analysis

The number of sessions required to achieve competency and demographic data and data on feeding practices was then entered directly into IBM SPSS version 22 (IBM Corp, Armonk, NY). Frequencies were tabulated and for continuous variables such as age, means and standard deviations were used. For the structured interviews, questions involving demonstration of a level of understanding were coded in terms of key words mentioned as described in Table 1. Thematic analysis of interview data was undertaken by examining the translated transcript and undertaking content analysis of answers and constructing a list of themes. HT and SM discussed each interview transcript and assigned themes to each answer/statement. For open-ended questions, peer counsellors were allowed to give as many examples as they wanted. Each example was assigned to a theme and the number of times this particular problem was separately raised was recorded.

**Table 1:** Coding for level of understanding of the project and the role of the peer counsellor

Comprehension score	Keywords required for Q1 Understanding of what the project is about	Keywords required for Q2 Understanding the role of the peer counsellors in the project
Limited understanding	Does not appropriately answer the question	Does not appropriately answer the question
Some understanding	Mentions Mirpur peer counselling project OR ward/cluster number	Mentions “peer counselling” or “Mirpur project”
Moderate understanding	Mentions one term in relation to breastfeeding promotion/infant feeding/colostrum/complementary feeding/child nutrition	Some understanding PLUS Mentions breastfeeding /infant feeding/child nutrition
High level of understanding	Moderate understanding PLUS Mentions information about correct dates when women start the counselling and when they complete it	Moderate understanding PLUS mentions problem solving OR being friendly and supportive to mothers OR motivation OR talking about helping women with position or attachment

## Results

The mean age of the peer counsellors was 31 years (95% Confidence Interval [CI] 27.5, 34.5), and the majority of the women (88%, 15/17) were married and all had received some form of schooling (Table 2). The education status of the peer counsellors was higher than aimed for initially, with 64% completing grades 8–10 and 35.3% completing grades 11–14. Among the 11 peer counsellors who had previously worked, 6 had worked in non-government organisations. The peer counsellors lived in small households with seven of the counsellors having one child and ten had 2 or 3 children.

**Table 2:** Socio-demographic characteristics of peer counsellors (*N* = 17)

Demographic variables		N	Mean or Percentage
Age	Mean (95% CI)	17	31.0 (27.5, 34.5)
	20–24	1	6
	25–29	9	53
	30+	7	41
Marital status	Married	15	88
	Separated/Widowed	2	12
Type of schooling	No schooling	0	0
	School and Madrasa	1	6
	School	16	94
Highest level of schooling (grade)	8–10	11	65
	11–14	6	35
Previous work	Yes	11	65
	No	6	35
Job	BRAC	3	
	Other NGO	3	
	Other	5	
Number of children	1	7	41
	2–3	10	59
Husbands age*	Mean (95% CI)	16	40 (34.6, 44.0)
Husbands schooling	School and Madrasa	3	
	School	13	
Highest level of schooling	Secondary	8	
	College or higher	8	
Number in the household	Mean (95% CI)		4.1 (3.3. 5.0)
	1–3	5	29
	4–6	11	65
	7–9	1	6
Household wealth index	Lower	2	12
	Middle	12	71
	Upper	3	18
Ever breastfed child?	Yes	17	100
How long after birth	Less than 1 h	12	71
	Within 1st day	4	24
	More than 1 day	1	6
Ever fed colostrum?	Yes	15	88
Still breastfeeding child	Yes	4	24
How old was the child when started on plain water	Mean age in months (95% CI)		5.4 (4.1, 6.6)
	< 4 months	5	29
	4–6 months	4	24
	➢ 6 months	8	47
Formula fed before 6 months	Yes	10	59
Complementary** feeding before 6 months	Yes	8	47
How old was the child when first started to give these foods to the child	Mean age in months (95% CI)		5.4 (4.1, 6.6)

*N = 16 (information on late husband was not provided by one peer counsellor)

**complementary foods include fresh milk, semolina, cerelac, khichuri, banana and other solid foods

CI: Confidence Interval

In terms of the peer counsellors’ history of feeding practices, 47.1% had followed the recommendations for > 6 months breastfeeding and a slightly higher proportion (52.9%) followed the recommendation for no solids before 6 months. A total of 41.2% of the peer counsellors had refrained from feeding baby formula or any other complementary food or milk before six months, compared to the Bangladesh Demographic and Health Survey percentage of 35.1% [8]. The average age of introduction of plain water and complementary foods was 5.35 months (95% CI 4.11, 6.60).

We also assessed the data on the number of supervised home visits needed for each peer counsellors to reach competency. On average the mean number of supervised home visits needed to reach competency was 16.9 (95% CI 12.3, 21.4). Education had a small impact on the number of visits required to reach competency. Those with higher education averaged 11.7 sessions compared to the 18.6 sessions averaged by the remaining peer counsellors who had completed some secondary education, although results were not significant (*p* = 0.171). No other demographic variables were associated with the number of visits need to achieve competency.

Information on structured interviews is given in Table 3. In the interviews, most women (82%, 14/17) understood what the project was about, having ‘some’ or ‘moderate’ understanding of the project, and one peer counsellors understanding of her role is illustrated in the following quote:

*“If a child grows up in the proper way that will help him/her to live a healthy life. We [peer counsellors] help mothers to feed their child the right food at the right age and I feel proud of it. I believe a healthy child can make a good nation.”*

**Table 3:** Results of structured interviews with peer counsellors (N = 17)

Question	Theme identified/comprehension score	N
Understanding of what the project is about*	Limited understanding	2
	Some understanding	6
	Moderate understanding	8
	High level understanding	1
Understanding of peer counsellors’ role in project*	Limited understanding	5
	Some understanding	6
	Moderate understanding	3
	High level understanding	3
Satisfied with job	Yes	17
	No	0
Number of daily visits performed	1	4
	2	11
	3	2
Perceived workload	I don’t have much (light)	9
	Average medium	6
	No mention	2
Happy with training they received	Yes	17
	No	0
Wants further training	Yes	13
	No	4
Felt they were able to deal with problems	Yes	2
	Mostly	15
	No	0
Steps taken when peer counsellors unsure of how to deal with problems	Discuss with supervisor	16
	No issues yet	1
Specific aspects of training that they found useful**	Benefits of colostrum	7
	Benefits and difficulties of exclusive breastfeeding	7
	Assisting with position and attachment	7
	Nutritious food for mother and child	3
	Iron tablets	3
	Antenatal care	2
	Disadvantages of formula feeding	2
	Procedure for complementary feeding	1
	Proper meal for age	1
What affects your ability to perform your job?**	Interruptions (phone rings, neighbours, older children interrupting during visits	7
	Mothers are too busy	6
	Mothers are kept quiet by in-laws	3
	Mothers not concentrating	2
	Mothers unhappy with messages	2
	Can’t make it to the birth within 3 days	1
	Limited by only giving nutrition information	1
	Peer counsellor very busy	1
	Child crying	1
	Mothers not home	1
Do peer counsellors see mothers outside visits	Yes	17
Peer counsellors developed a close friendship with mothers	Yes	17
How do you deal with obstacles within the mothers family during the peer counselling session**	Try and motivate family members	16
	Enlist assistance from supervising peer counsellor	4
	Try and correct misconceptions	1
Peer counsellors happy with incentives given	Yes	5
	Would appreciate an increment in salary	11
	No	1

*see Table 1 for explanation of how coding was developed **questions presented as open-ended, number represents the number of peer counsellor who raised this issue

There was a high level of job satisfaction within the group, with all 17 peer counsellors saying they were happy working for the project. This is shown in the following quote:

*“I am very happy working on this project. I am also very happy to learn many things and make mothers understand the benefits of visits. Mothers also cordially receive messages and are well behaved with me.”*

All peer counsellors expressed satisfaction with the training they had received, while a majority (76%, 13/17) said they would like to have further training. All 17 peer counsellors reported that they saw the mothers they counselled outside scheduled visits and some had developed close friendships with these women.

In terms of the work the peer counsellors completed every day, 11 of the peer counsellors performed 2 visits per day (approximately one hour each visit), with 9 perceiving their workload to be ‘not too much’, 6 describing that they had an ‘average or medium workload’ and 2 peer counsellors did not comment on how they found the workload.

There were broad responses in terms of which aspect of training the peer counsellors found most useful, with the most reported answers being ‘the benefits of colostrum’, ‘the benefits and difficulties of exclusive breastfeeding’, and ‘positioning and attachment’.

The women faced various challenges whilst trying to complete their daily tasks and many were dismayed when mothers did not follow the breastfeeding guidelines they were given. In the following quote for example, one peer counsellor expressed disappointment when, despite her best effort, mothers still chose to formula feed.

*“I am feeling unhappy when mothers go for infant formula instead of breastmilk. I tried my best to motivate[them] but some mothers are not of the same mind.”*

Interruptions, as well as mothers being too busy were the most highly reported occurrences that reduced their ability to perform their job (Table 3). ‘Interruptions’ were mentioned by 7 women and ‘mothers being too busy’ by 6 women. In the following quote one peer counsellor describes the challenges of mothers being too busy:

*“Sometimes mothers are very busy, they don’t have enough time to feed their baby the way we demonstrate. These matters affect my ability to carry out my job.”*

Another barrier experienced by the women was ‘mothers being kept quiet by in-laws’, this was mentioned by 3 of the peer counsellors and the following quote captures this and illustrates one of the strategies used to deal with the problem:

*“Sometimes other family members create interruptions during counselling sessions. I try and motivate them [about feeding]. If I am not successful that day I visit the mother again on another day and try and motivate them again.”*

## Discussion

This study documents the experiences of peer counsellors delivering infant feeding information and support to women in urban Dhaka and shows all were satisfied with their role and most had developed close relationships with the mothers they visited. This is a positive outcome of the project as evidence shows the rapport established in mother-to-mother support is a basis for mothers to breastfeed exclusively for longer [14, 21]. However, the intervention was not without challenges for the peer counsellors. Interruptions, as well as mothers being too busy were the most highly reported occurrences that reduced the peer counsellors’ ability to perform their job. The education status of the peer counsellors may have had a small effect on reaching the competency standard in the shortest time.

The success of the peer-to-peer relationship in ensuring mothers are best supported appears to be very important. Part of this connection may be the personal relationships and rapport built with face-to-face contact. In a study conducted in Malaysia, professional lactation consultants providing telephone-based support to mothers was shown to be ineffective at increasing breastfeeding prevalence at 4 and 6 months [17]. A Cochrane review of breastfeeding supports found that trials without face-to-face contact, or when women had to initiate support sessions, were likely to be less effective [21]. In this study the schedule of home visits was initiated by the peer counsellors. The strong bonds that peer counsellors are developing with mothers is an early indicator of success, however this can only be fully discussed within the context of the final study outcomes.

Despite several government and non-government infant and young child feeding programmes using community health workers to support media messages on breastfeeding since 2007, there are still strong cultural and family pressures on mothers in Bangladesh to mix feed [22]. The proportion of mothers feeding prelacteal foods (foods such as glucose water or honey) to their babies was reported at 39% in the Bangladesh Demographic and Health Survey [8]. This has also been reported from peer supporters who worked in the PROMISE-EBF study [20]. In focus group discussions in South Africa, women explained how mothers would often agree with their recommendations but continue feeding very young children complementary foods, and consequently the peer counsellors felt disheartened when a mother mixed-fed her infant [20]. The study also reported that they felt very powerless in these situations. Similarly, in our study, peer counsellors expressed disappointment when mothers chose to mixed feed their infants.

In our study 16 of 17 peer counsellors said they would discuss a problem with their supervisor if they were unsure how to deal with it. Peer supporters in the PROMISE-EBF trial similarly reported that the supervisors were seen as a resource they could use for assistance in difficult situations [20]. The authors of that trial recommended that further support needed to be provided to peer counsellors not only to deal with technical difficulties but also to be responsive to the challenges and emotional strains faced by the women in the process of delivering the intervention [20]. Community health worker projects, including those using peer counsellors, have previously been criticised for the lack of support they provide for the community workers [23]. It is important to provide adequate support and perhaps forums for the peer counsellors to share negative experiences, because over time this may burden and dishearten the counsellors. In other studies focus groups have been used for peer counsellors to voice their concerns [20]. This is also reflected in our study where the majority of peer counsellors indicated that they would like further training particularly around motivating busy mothers to follow breastfeeding recommendations and dealing with interruptions and unsupportive family members.

If the intervention is found to be successful in improving breastfeeding, and deemed suitable to scale up, the peer counsellors’ role could be expanded to include a broader range of health issues. One peer counsellor in our study mentioned that it may be helpful if the peer counsellors were trained in a range of child and maternal health topics and were able to offer services such as family planning and advice and support in accessing relevant services.

### Strengths and limitations

One of the main strengths of this study was that all the peer counsellors were included in this research, meaning that all experiences of being part of this trial have been recorded and presented. Another advantage was a mix of quantitative and qualitative data collection methods, enabling us to present a more complete picture of the experiences of the peer counsellors.

Limitations include the small number of peer counsellors, which limited our ability to find patterns and trends using demographic associations. In terms of structured interview methodology, questions assessing understanding of how much the peer counsellors understood of the project may have been misclassified because keywords were not mentioned. Hence the results of this approach should be interpreted with caution. Also, social desirability bias may have affected the responses of the peer counsellors describing the ways they had fed their own babies. The questionnaire was completed once women had already received training. Some peer counsellors may have felt embarrassed to share negative experiences or failures in feeding, despite understanding the importance of nutrition and breastfeeding in early infancy. Nevertheless, rates of breastfeeding in the peer counsellors were comparable with those described in Bangladesh Demographic and Health Survey [8] and so it is reasonable to conclude that this effect was minimal.

Similarly, bias may have come into play when the interviews were conducted by the project manager. The women may have wanted to please her and altered their answers so as to underrepresent challenges they were experiencing in their role.

### Recommendations for future practice

We recommend that in future projects including peer counselling for breastfeeding, more focus should be placed on the communications aspect of training, to assist women in dealing with non-supportive family members and managing interruptions effectively. There is potential for implementing focus groups to assist peer counsellors in troubleshooting similar problems they are experiencing. Focus groups could also be used to collect more detailed research about the barriers the peer counsellors are facing. Focus groups have been used in many studies, such as the study by Nkonki and Daniels in South Africa [20] to record women’s experiences in a detailed and thematic way.

Interviews in the future should be conducted by an external source, so the peer counsellors would feel more comfortable expressing concerns they felt about the study.

Our study suggests there was a trend toward more educated peer counsellors needing less training to achieve competency and this may assist in reducing training costs. However, it is crucial to be able to keep peer counsellors as ‘true peers’ who can effectively work on the same level as the mothers they are supporting.

## Conclusions

This study adds to the literature documenting the experiences of peer counsellors working to support breastfeeding mothers. Our study found that peer counsellors were satisfied with their role and with the training that they received, and most felt that they were able to deal with common breastfeeding problems. Most of the peer counsellors felt they needed further training in order to overcome challenges such as dealing with non-supportive family members and interruptions. The study provides valuable data to improve implementation of future peer counselling strategies.

## Supplementary information

**Additional file 1.** Tool for monitoring performance of peer counsellors.

**Additional file 2.** Structured interview questions for peer counsellors.

## Data Availability

Data can be requested from the lead author and will be supplied in de-identified form.
