# Supplementary material for: Characteristics and experiences of peer counsellors in urban Dhaka: a structured interview study
Source: Int Breastfeed J. 2019 Nov 6;14:48. doi: 10.1186/s13006-019-0240-y (PMC6836400; doi:10.1186/s13006-019-0240-y)
Supplement: Supplementary file 1 — Additional file 1. Tool for monitoring performance of peer counsellors. [file 13006_2019_240_MOESM1_ESM.docx]

**Additional file 1**

**Tool for monitoring performance of peer counsellors**

This Peer Counsellor Monitoring Form should be used by the Senior Breastfeeding Counsellor to monitor the work performance of the Peer Counsellor

Peer counsellors should:

1. Use culturally appropriate greeting and gestures that convey respect and caring
2. Clearly explain the objective of the visit
3. Create a comfortable atmosphere for woman to ask question
4. Ensure privacy and confidentiality
5. Use open questions to identify mother’s concern
6. Show empathy for good progress and for problems experienced
7. Enquire about infant feeding status (probed)
8. Check positioning of the baby
9. Explain the relevant information for infant feeding at this stage
10. Use probing questions to clarify mother’s actual practices and specific problems
11. Clarify misconceptions, if they are observed
12. Encourage mothers to talk freely and to think of own solutions while providing expert advice where needed
13. Encourage mother for frequent and demand breastfeeding
14. Message given to the mother/family members regarding food and rest
15. Message given to the mother/family members regarding benefits of exclusive breastfeeding

For each question peer counsellors are rated on the scale below and each practice must score 4 or above to achieve competency.

| **1** | **None:** Does not perform adequately, should be observed for subsequent session |
| --- | --- |
| **2** | **Limited**: Very limited strength or skill; needs close support from the observer |
| **3** | **Some:** Some ability or skill in this area but needs more practice |
| **4** | **Strong:** Strong skills/strength in this area; but could work on a few critical areas |
| **5** | **Excellent:** excellent skill; ready to counsel independently |
