# Supplementary material for: Characteristics and experiences of peer counsellors in urban Dhaka: a structured interview study
Source: Int Breastfeed J. 2019 Nov 6;14:48. doi: 10.1186/s13006-019-0240-y (PMC6836400; doi:10.1186/s13006-019-0240-y)
Supplement: Supplementary file 2 — Additional file 2. Structured interview questions for peer counsellors. [file 13006_2019_240_MOESM2_ESM.docx]

**Additional file 2**

**Structured interview questions for peer counsellors**

1. What is your understanding of what the project is about?*
2. What is your understanding of peer counsellors role in project?*
3. Are you satisfied with job? Would you like to elaborate?
4. About how many visits do you perform every day?
5. How do you find your workload ?
6. Are you happy with the training you received?
7. What are the specific aspects of training that you found most useful?
8. Do you want to go for further training?
9. Do you feel you are able to deal with most problems faced by the mothers?
10. What are the steps you take when you are unsure of how to deal with problems?
11. What affects your ability to perform your job?
12. Do you see the mothers outside visits?
13. How do you deal with obstacles within the mother’s family during the peer counselling session?
14. Are you happy with your salary/incentives given?

*see Table 1 for explanation of how coding was developed
